# Optimizing the phenotyping of rodent ASD models: enrichment analysis of mouse and human neurobiological phenotypes associated with high-risk autism genes identifies morphological, electrophysiological, neurological, and behavioral features

**DOI:** 10.1186/2040-2392-3-1

**Published:** 2012-02-20

**Authors:** Joseph D Buxbaum, Catalina Betancur, Ozlem Bozdagi, Nate P Dorr, Gregory A Elder, Patrick R Hof

**Affiliations:** 1 Seaver Autism Center for Research and Treatment, Mount Sinai School of Medicine, One Gustave L Levy Place, New York, NY 10029, USA; 2 Department of Psychiatry, Mount Sinai School of Medicine, One Gustave L Levy Place, New York, NY 10029, USA; 3 Department of Neuroscience, Mount Sinai School of Medicine, One Gustave L Levy Place, New York, NY 10029, USA; 4 Department of Genetics and Genomic Sciences, Mount Sinai School of Medicine, One Gustave L Levy Place, New York, NY 10029, USA; 5 Friedman Brain Institute, Mount Sinai School of Medicine, One Gustave L Levy Place, New York, NY 10029, USA; 6 Department of Neurology, Mount Sinai School of Medicine, One Gustave L Levy Place, New York, NY 10029, USA; 7 Inserm U952, 9 quai Saint Bernard, 75005 Paris, France; 8 CNRS UMR 7224, 9 quai Saint Bernard, 75005 Paris, France; 9 UPMC Univ Paris 06, 9 quai Saint Bernard, 75005 Paris, France; 10 Neurology Service, James J. Peters VA Medical Center, Bronx, NY 10468, USA

**Keywords:** Systems biology, mouse behavior, autism, autism spectrum disorders, genetically modified mice, forward genetics, reverse genetics

## Abstract

**Background:**

There is interest in defining mouse neurobiological phenotypes useful for studying autism spectrum disorders (ASD) in both forward and reverse genetic approaches. A recurrent focus has been on high-order behavioral analyses, including learning and memory paradigms and social paradigms. However, well-studied mouse models, including for example *Fmr1 *knockout mice, do not show dramatic deficits in such high-order phenotypes, raising a question as to what constitutes useful phenotypes in ASD models.

**Methods:**

To address this, we made use of a list of 112 disease genes etiologically involved in ASD to survey, on a large scale and with unbiased methods as well as expert review, phenotypes associated with a targeted disruption of these genes in mice, using the Mammalian Phenotype Ontology database. In addition, we compared the results with similar analyses for human phenotypes.

**Findings:**

We observed four classes of neurobiological phenotypes associated with disruption of a large proportion of ASD genes, including: (1) Changes in brain and neuronal morphology; (2) electrophysiological changes; (3) neurological changes; and (4) higher-order behavioral changes. Alterations in brain and neuronal morphology represent quantitative measures that can be more widely adopted in models of ASD to understand cellular and network changes. Interestingly, the electrophysiological changes differed across different genes, indicating that excitation/inhibition imbalance hypotheses for ASD would either have to be so non-specific as to be not falsifiable, or, if specific, would not be supported by the data. Finally, it was significant that in analyses of both mouse and human databases, many of the behavioral alterations were neurological changes, encompassing sensory alterations, motor abnormalities, and seizures, as opposed to higher-order behavioral changes in learning and memory and social behavior paradigms.

**Conclusions:**

The results indicated that mutations in ASD genes result in defined groups of changes in mouse models and support a broad neurobiological approach to phenotyping rodent models for ASD, with a focus on biochemistry and molecular biology, brain and neuronal morphology, and electrophysiology, as well as both neurological and additional behavioral analyses. Analysis of human phenotypes associated with these genes reinforced these conclusions, supporting face validity for these approaches to phenotyping of ASD models. Such phenotyping is consistent with the successes in *Fmr1 *knockout mice, in which morphological changes recapitulated human findings and electrophysiological deficits resulted in molecular insights that have since led to clinical trials. We propose both broad domains and, based on expert review of more than 50 publications in each of the four neurobiological domains, specific tests to be applied to rodent models of ASD.

## Findings

### Approach

There is overwhelming evidence for a critical role for genetic variation in autism spectrum disorders (ASD) [1,2]. Moreover, all of the genes and loci reliably identified to date are associated with significant risk, such that genetically modified rodents recapitulating an ASD gene defect will have strong construct validity for ASD. There have been many such models created and we can now ask which neurobiological domains are altered in a recurrent manner when an ASD gene is disrupted. The observed mouse phenotypes can then be related to those observed in humans with a similar gene disruption to assess face validity of rodent models for ASD.

In order to achieve these goals, we made use of manually curated lists of genes implicated in ASD. In contrast to other studies, we focused on genes where there was prior evidence of an etiological role in ASD (that is, genes of major effect for ASD). We began with a carefully curated list of 103 such genes implicated in ASD, with or without intellectual disability (ID), from a recent review by one of us (CB) [3]. In that study, an extensive literature search was conducted looking for articles describing genetic disorders in patients with autism, ASD, pervasive developmental disorder, Asperger syndrome, or autistic/autistic-like traits/features/behavior, using PubMed and Google Scholar, as well as follow-up of references cited in the papers thus identified. This list has been routinely updated by the author using the same criteria, and nine additional genes were added to the published report (*DPYD, FOLR1, GNS, GRIN2B, HEPACAM, HGSNAT, KCNJ11, NAGLU*, and *STXBP1*). The final list of 112 genes implicated in ASD (ASD112) is shown in Additional file 1. Since most high-risk ASD genes were identified by unbiased genetic approaches (for example, characterization of translocation breakpoints, recurrent copy number variants, X-linked genes first identified by linkage, and so on), ASD112 represents a largely unbiased list of such genes. As part of our analyses we made use of three prior publications to identify 31 genes from the 112 that were identified in large-scale proteomic analyses of human or mouse postsynaptic densities, or mouse presynaptic or postsynaptic proteomes [4-6]. These 31 genes are referred to as synaptic ASD genes (ASD31 in additional file 1). While there are likely additional synaptic genes in ASD112 that were not included in ASD31 (for example, *NLGN4X*), we chose to identify synaptic genes using empirical proteomic datasets such that this list would remain unbiased by genes chosen for further detailed study.

We then took these two ASD gene lists and used them to identify significantly enriched categories of mouse and human phenotypes from the Mammalian Phenotype Ontology (MPO) project [7], using ToppGene [8]. We used the hypergeometric test to exactly calculate *P *values for enrichment and further required that the Bonferroni corrected *P *value be < 0.01. We made use of the standard hypergeometric approach defining the hypergeometric probability, h(x; N, n, k), as {[_k_C_x_][_N-k_C_n-x_]}/[_N_C_n_], where N is the number of annotated genes in the genome, k is the number of genes in the genome that are associated with a given phenotype or other category, n is the number of genes in the test list (for example, ASD112), x is the number of genes in the test list that have the given phenotype or other category, and _k_C_x _represents the number of combinations of k things, taken x at a time. This approach ensures that the findings we describe are quantitative and statistically substantiated, while also being conservative (as we use an analysis-wide Bonferroni correction).

### Enriched mouse phenotype (MP) categories

**Figure 1:**
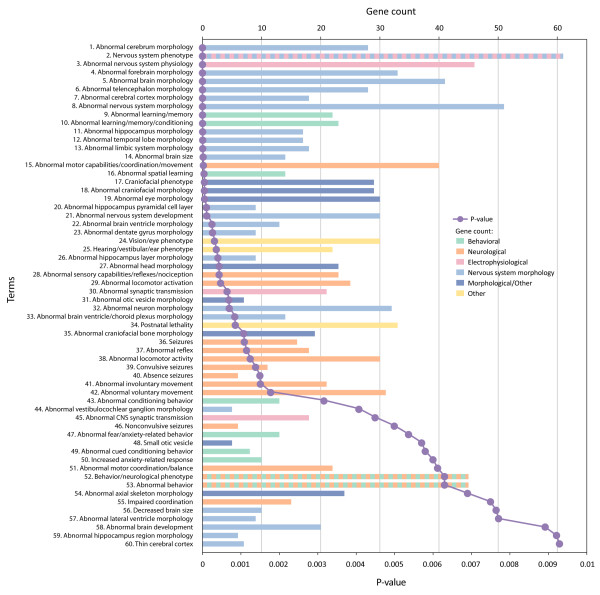
**Mouse phenotype categories associated with ASD genes**. ASD genes (n = 112) were analyzed for enrichment in mouse phenotypes using ToppGene with a Bonferroni corrected *P *value cutoff of 0.01. Categories are arranged from most significant and downwards (purple line), and for each category, the number of genes in the ASD112 list for which there were murine models with the associated category are indicted by the length of the horizontal bars (gene count). To highlight differing phenotypic categories discussed in the text, bars are color-coded as indicated in the inset to the figure. Categories relating to nervous system morphology phenotype domains are colored light blue, while other morphological categories are colored dark blue, electrophysiological categories are colored pink, neurological categories are colored peach, and higher-order behavioral categories are colored green. Categories corresponding to more than one phenotyping domain are presented as alternating colors, and categories that do not relate to our phenotyping scheme are colored yellow. All data are also found in tabular form in Additional file 2.

We asked whether known ASD disease genes could be used to provide insights into defined murine phenotypes. Using the entire ASD gene list, we identified 60 categories significantly enriched in the mouse phenotype categories after correction (Figure 1 and Additional file 2). Of the 60 categories, 23 were directly related to alterations in brain or neuronal morphology (note the coloration of bars in Figure 1), with an additional eight categories associated with other morphological changes. A further 21 categories related to changes in observable behaviors. However, most behavioral findings reflected alterations in neurological processes (MP categories, from lowest *P *value and up were: MP:0002066, abnormal motor capabilities/coordination/movement; MP:000206, abnormal sensory capabilities/reflexes/nociception; MP:0003313 abnormal locomotor activation; MP:0002064, seizures; MP:0001961, abnormal reflex; MP:0001392, abnormal locomotor activity; MP:0000947, convulsive seizures; MP:0003216, absence seizures; MP:0003492, abnormal involuntary movement; MP:0003491, abnormal voluntary movement; MP:0001516, abnormal motor coordination/balance; MP:0001405, impaired coordination) with a smaller number reflecting deficits in high-order processes (MP:0001449, abnormal learning/memory; MP:0002063, abnormal learning/memory/conditioning; MP:0001463, abnormal spatial learning; MP:0002062, abnormal conditioning behavior; MP:0002065, abnormal fear/anxiety-related behavior; MP:0001454, abnormal cued conditioning behavior; MP:0001363, increased anxiety-related response; MP:0004924, abnormal behavior).

Three enriched terms were related to electrophysiology and neuronal transmission (MP:0003635, abnormal synaptic transmission; MP:0003633, abnormal nervous system physiology; and MP:0002206, abnormal CNS synaptic transmission) providing further objective support for the important role of electrophysiological analyses in understanding pathophysiology in ASD. Note that there were over 40 genes associated with changes in electrophysiology and neuronal transmission (see below) and that these changes were quite diverse, including enhanced hippocampal long-term potentiation and long-term depression as well as deficits or no changes in these measures.

Related to the electrophysiological deficits were the categories associated with seizure disorders (MP:0002064, seizures; MP:0000947, convulsive seizures; MP:0003216, absence seizures; MP:0000948, non-convulsive seizures) and sensory and motor abnormalities (MP:0002066, abnormal motor capabilities/coordination/movement; MP:000206, abnormal sensory capabilities/reflexes/nociception; MP:0003313, abnormal locomotor activation; MP:0001961, abnormal reflex; MP:0001392, abnormal locomotor activity; MP:0003492, abnormal involuntary movement; MP:0003491, abnormal voluntary movement; MP:0001516, abnormal motor coordination/balance; MP:0001405, impaired coordination).

**Figure 2:**
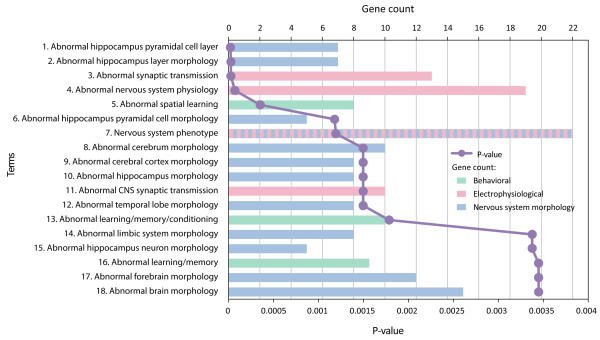
**Mouse phenotype categories associated with synaptic ASD genes**. Thirty-one ASD genes (ASD31) found in the synaptic proteome were analyzed for enrichment in mouse phenotypes using ToppGene with a Bonferonni corrected *P *value cutoff of 0.01. Further details regarding the figure and inset are described in the legend to Figure 1. All data are also found in tabular form in Additional file 3.

Similar conclusions can be made from the synaptic ASD genes (Figure 2 and Additional file 3). Eleven out of the eighteen significant terms related to alterations in brain and neuronal structure, three terms related to electrophysiology and synaptic transmission (MP:0003635, abnormal synaptic transmission; MP:0003633, abnormal nervous system physiology; and, MP:0002206, abnormal CNS synaptic transmission) and three terms related to behavioral changes (MP:0001463, abnormal spatial learning; MP:0002063, abnormal learning/memory/conditioning; and, MP:0001449, abnormal learning/memory).

### Enriched human phenotype (HP) categories

**Figure 3:**
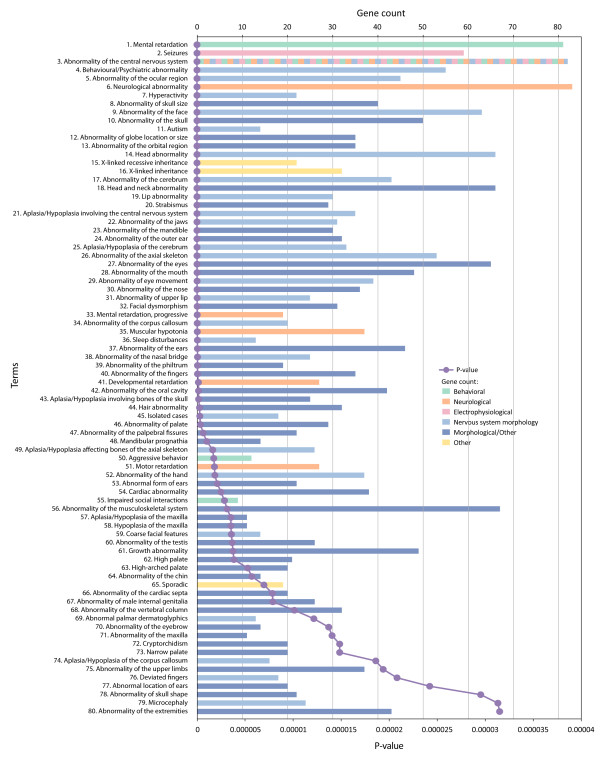
**Human phenotype categories associated with ASD genes**. ASD genes (n = 112) were analyzed for enrichment in human phenotypes using ToppGene with a Bonferonni corrected *P *value cutoff of 0.01. This figure only shows the first 80 categories ranked from most significant *P *value; the full list of 151 significant categories is found in tabular form in Additional file 4. Further details regarding the figure and inset are described in the legend to Figure 1.

One can also ask what human phenotypes are enriched with these ASD genes. Although the genes have been collated because of their role in ASD, an unbiased view can provide an assessment of the fuller spectrum of associated phenotypes as well as the relationship of these phenotypes to the enriched mouse phenotypes. We identified 151 significantly enriched categories (Additional file 4); the top 80 are shown in Figure 3. Interestingly, we observed significant overlaps in human and mouse phenotypes associated with ASD genes. Using the full ASD gene list, there was direct evidence for changes in brain morphology (HP:0002060, abnormality of the cerebrum; HP:0002977, aplasia/hypoplasia involving the central nervous system; HP:0007364, aplasia/hypoplasia of the cerebrum; HP:0001273, abnormality of the corpus callosum; HP:0007370, aplasia/hypoplasia of the corpus callosum; HP:0000252, microcephaly; HP:0000256, macrocephaly; HP:0002118, abnormality of the cerebral ventricles; HP:0002119, ventriculomegaly; HP:0001339, lissencephaly; HP:0002059, cerebral atrophy; HP:0007369, atrophy/degeneration affecting the cerebrum). There was also indirect evidence for alterations in brain morphology given the very extensive numbers of categories affecting craniofacial morphology.

Electrophysiological deficits are not systematically assessed in patients with ASD, but there was evidence for such changes associated with many ASD genes (HP:0001311, neurophysiological abnormality; HP:0001250, seizures; HP:0002353, EEG abnormalities). Furthermore, there was extensive evidence for neurological abnormalities in these analyses. In addition to the brain morphology and physiological abnormalities noted above, significant categories included: HP:0000707, neurological abnormality; HP:0000496, abnormality of eye movement; HP:0001252, muscular hypotonia; and, HP:0001270, motor retardation. Behavior findings included: HP:0001249, mental retardation; HP:0000752, hyperactivity; HP:0000717, autism; HP:0006887, mental retardation, progressive; HP:0002360, sleep disturbances; HP:0000718, aggressive behavior; HP:0000735, impaired social interactions; HP:0002117, speech delay; HP:0002116, deficiency of speech development; HP:0000733, stereotyped, repetitive behavior; HP:0002456, severe behavioral problems at age 3 to 4 years; HP:0000748, inappropriate laughter; and HP:0000749, paroxysmal bursts of laughter.

### Implications for phenotyping rodent models for ASD

Based on our analyses, we propose five broad categories of analysis for rodent models of ASD, including biochemical and molecular analyses using both targeted and genome-level approaches (which are not captured in the MP databases) and the four domains we identify in our analyses (Table 1). We can further assess specific relevant assays for ASD rodent models by reviewing findings to date. To do this, we took the top 10 (based on *P *values) categories for morphology (Additional file 5) or behavior (Additional file 6), and all three top categories for electrophysiology (Additional file 7), and surveyed the literature on phenotypes in mice with a targeted disruption of the gene.

**Table 1:** Recommended assays for ASD-related changes in rodent models

Levels of analysis	Specific assays
Molecular assays	Focused biochemical and molecular assays directed at the targeted gene and pathway Unbiased proteomic and genome-wide gene expression analysis, with pathway and network analysis
Nervous system morphology assays	Gross pathology Analyses of neuronal fate and neuronal migration including tract-tracing Assessment of neuronal morphology, dendritic and spine integrity, and synaptic morphology
Electrophysiological assays	Use of evoked field potentials to assess basal synaptic properties, short-term plasticity, long-term plasticity, and analysis of seizure-like activity Patch-clamp recording for basic membrane properties, characterization of voltage- and ligand-gated ion channel currents, and assessment of spontaneous neurotransmission
Neurological assays	General observations, spontaneous and elicited behaviors Assessment of grip strength, sensitivity to pain, gait, accelerating rotarod, acoustic startle, and prepulse inhibition of acoustic startle Analysis of seizure susceptibility
Higher-order behavioral assays	Assessment of anxiety, open field/spontaneous locomotor behavior Analysis of social interaction/recognition, including ultrasonic vocalizations Assessment of learning and memory, including cued and contextual fear conditioning, Morris water maze, radial arm maze, and passive avoidance Recording of behavioral stereotypes

Based on a survey of findings, we conclude that five levels of analysis should be carried out on rodent ASD models. Four are derived from the unbiased enrichment analyses described in the main text and the fifth (molecular) is part of the standard and enhanced workup of novel model systems. In each of the five domains, specific assays are suggested, based on expert review of publications of at least 40 genes associated with electrophysiological, neurological, or higher behavioral features.

Examining ASD genes associated with morphological changes, we can see that brain abnormalities occur generally among three major categories: (1) major developmental pathologies of the neuraxis (13 genes); (2) alterations in the development of specific regions of the brain with abnormal migration of neurons as well as glial cells (15 genes); and (3) alteration of neuronal morphologies and anomalies of dendritic spines and synapses (21 genes). The morphologic characterization of the first category of abnormalities may lend itself to simple analyses of gross pathology as well as magnetic resonance microscopy, while the second and third categories would involve quantitative analyses of neuronal fate and neuronal migration at the level of specific populations of precursors, tract-tracing using diffusion tensor imaging, and assessment of neuronal morphology, dendritic and spine integrity at the single spine level of resolution, and electron microscopy of synaptic morphology and distribution of specific synaptic markers using immunoelectron microscopy methods. The data obtained by such approaches can then be correlated to electrophysiological and behavioral data.

Electrophysiological findings in mouse models amongst the over 40 ASD genes surveyed fell into two broad areas: (1) changes in evoked field potentials measured by extracellular recording in brain slices; and (2) changes in ion channel function measured by patch-clamp recordings in brain slices. Brain regions examined were hippocampus, neocortex, cerebellum, and amygdala. For the evoked potentials, analyses included those for basal synaptic properties, short-term plasticity (using the paired-pulse paradigm), long-term plasticity (with induction of long-term potentiation by high-frequency or theta-burst stimulation, or induction of long-term depression by low-frequency stimulation), and seizure-like activity (using low Mg^2+^). Patch-clamp recordings focused on basic membrane properties, characterization of voltage-gated (Na^+^, K^+^, Ca^2+^) and ligand-gated (NMDA, AMPA, GABA) ion channel currents, and assessment of spontaneous neurotransmission (spontaneous miniature inhibitory and excitatory events by measuring inhibitory and excitatory post-synaptic currents).

Behavioral analyses were quite diverse in the papers reviewed but, based on analysis of positive findings, we can propose the following. For neurological changes, observing spontaneous and elicited behaviors with protocols such as the SHIRPA protocol are appropriate, including an assessment of grip strength, sensitivity to pain (for example, tail flick), gait analysis, accelerating rotarod, acoustic startle, prepulse inhibition of acoustic startle, and seizure susceptibility (spontaneous, drug-induced, auditory evoked). For high-order behaviors, we would include assessment of anxiety (for example, elevated zero maze and open field), spontaneous locomotor behavior, social interaction/recognition, cued and contextual fear conditioning, Morris water maze, radial arm maze, passive avoidance, observation for stereotyped behaviors, and ultrasonic vocalizations. These recommendations are consistent with other careful analyses of mouse behaviors relevant to ASD [9].

## Conclusions

ASD are defined by alterations in a triad of symptom domains, including impairments in social interactions, impairments in language or the social use of language, and by restricted, repetitive, and stereotyped patterns of behaviors and interests. A significant challenge to analyzing ASD in humans is the restricted tools available for studying the human brain. For this reason, animal models provide a critical means to understand pathophysiology and identify therapeutic opportunities. As noted above, because there are known, high-risk, ASD genes, rodent model systems can be developed with strong construct validity for ASD. As we begin to assess novel therapeutics in ASD, determining which phenotypes in rodents have face and ultimately predictive validity in ASD becomes an important aim. Many attempts have been made to describe rodent phenotypes that fall within the ASD triad in order to establish face validity. This study differs from such recent studies in that rather than asking what behaviors in rodents might reflect the core features of ASD, we asked what phenotypes in rodents were observed in mice with disruptions in diverse ASD causal genes. This provides an unbiased assessment of ASD phenotypes associated with these genes. We also carried out a similar, unbiased analysis of phenotypes in patients with disruptions in ASD genes and showed that the categories outside of the core ASD behavioral triad were similarly impacted in both the mouse and human analyses. This provides important face validity to the analyses.

In spite of the fact that high-risk ASD genes analyzed in the current report impact a large number of pathways (Additional file 8), an unbiased assessment of murine phenotypes associated with ASD genes shows clear evidence for effects on brain and neuronal morphology, electrophysiology, neurological features, and high-order behavior. In addition, the analyses indicate that behavioral deficits in ASD models are frequently not in social domains, supporting a broad examination of neurological as well as high-order behavioral paradigms to support a neurodevelopmental function for the gene under study. It also suggests that forward genetics, starting from a deficit in high-order behaviors, may obscure many important genes and pathways. Electrophysiological changes differed across different ASD genes and in differing directions, indicating that the excitation/inhibition imbalance hypothesis is not specific or is too broad as to be not falsifiable.

Caveats with the current approach are that not all analyses have been applied to all mouse models and even if similar approaches were used they were not always identically applied. Moreover, we did not explicitly catalog models with disruptions in high-risk ASD genes where specific phenotypes were not observed. Finally, the MPO database is incomplete and biases might be introduced by choices of genes to study and disrupt, and by phenotyping choices.

One can argue that amongst the four broad categories identified in unbiased analyses, morphological and electrophysiological alterations are most proximal to changes in gene expression, and may then impact on seizure risk and alterations in sensory and motor function, followed by effects on high-order behaviors. In this view, a focus on low-order processes may be more informative than a focus on high-order processes. This is a departure from recent recommendations in that we conclude that the data argue for a strong bottom-up approach to phenotyping rodent models of ASD. Certainly, in the case of several ongoing clinical trials for monogenic forms of ASD, including Fragile X syndrome, tuberous sclerosis, and Rett syndrome, molecular, morphological, and electrophysiological analyses were key parts of target identification and even preclinical studies, as were behavioral phenotypes.

Altogether, these results highlight how basic neuroscience approaches in model systems identify relevant and important phenotypes that will continue to shed light on ASD pathophysiology at multiple levels.

## Abbreviations

ASD: autism spectrum disorders; CNS: central nervous system; HP: human phenotype; ID: intellectual disability; MP: mouse phenotype; MPO: Mammalian Phenotype Ontology.

## Competing interests

The authors declare that they have no competing interests.

## Authors' contributions

JDB, CB, OB, NPD, GAE, and PRH were involved in the conception of the study and in the editing of the manuscript. CB developed the gene lists. JDB carried out the enrichment analyses and prepared the first draft of the manuscript. Expert review of prior publications was carried out for morphology (PRH), electrophysiology (OB), and behavior (NPD and GAE). All authors read and approved the final manuscript.

## Supplementary Material

Additional file 1 **Gene lists**. Excel file containing the lists of the 112 ASD genes and the 31 synaptic ASD genes. Click here for file

Additional file 2 **Mouse phenotype categories associated with ASD genes**. Excel file with the enrichment analysis of mouse phenotypes using as the seed list 112 ASD genes (Additional file 1). Columns are mouse phenotype (MP) ID, MP name, enrichment *P *value, Term in Query, and Term in Genome (number of genes in the murine genome that were associated with that MP category at the time of the analysis). Click here for file

Additional file 3 **Mouse phenotype categories associated with synaptic ASD genes**. Excel file with enrichment analysis of mouse phenotypes using as the seed list 31 synaptic ASD genes (Additional file 1). See legend for Additional file 2 for explanation of column headers. Click here for file

Additional file 4 **Human phenotype categories associated with ASD genes**. Excel file with enrichment analysis of human phenotypes using as the seed list 112 ASD disease genes (Additional file 1). Columns are human phenotype (HP) ID, HP name, enrichment *P *value, Term in Query and Term in Genome. See legend for Additional file 2 for explanation of column headers. Click here for file

Additional file 5 **Genes in the top 10 mouse phenotype morphology categories**. Excel file with the genes found in the top 10 categories relating to morphological abnormalities associated with mouse mutations in ASD genes. The seed list used was the 112 ASD genes. For each category, columns indicate Entrez Gene ID, Gene Symbol, and Gene Name. Click here for file

Additional file 6 **Genes in the top 10 mouse phenotype behavioral categories**. Excel file with the genes found in the top 10 categories relating to behavioral abnormalities associated with mouse mutations in ASD genes. See Additional file 5 legend for more information. Click here for file

Additional file 7 **Genes in the top mouse phenotype electrophysiological categories**. Excel file with the genes found in the top (n = 3) categories relating to electrophysiological abnormalities associated with mouse mutations in ASD genes. See Additional file 5 legend for more information. Click here for file

Additional file 8 **Gene Ontology categories associated with ASD genes**. Excel file with the enrichment analysis of Gene Ontology (GO) categories using as the seed list 112 ASD genes (Additional file 1). Columns are GO, GO name, enrichment *P *value, Term in Query, and Term in Genome. Click here for file
